# A multi-timescale synaptic weight based on ferroelectric hafnium zirconium oxide

**DOI:** 10.1038/s43246-023-00342-x

**Published:** 2023-02-17

**Authors:** Mattia Halter, Laura Bégon-Lours, Marilyne Sousa, Youri Popoff, Ute Drechsler, Valeria Bragaglia, Bert Jan Offrein

**Affiliations:** 1 IBM Research Europe - Zurich Research Laboratory, CH-8803 Rüschlikon, Switzerland; 2 ETH Zurich - Integrated Systems Laboratory, CH-8092 Zurich, Switzerland; 3 Present Address: Lumiphase AG, CH-8712 Stäfa, Switzerland

**Keywords:** Electronic devices, Electronic devices, Computational science

## Abstract

Brain-inspired computing emerged as a forefront technology to harness the growing amount of data generated in an increasingly connected society. The complex dynamics involving short- and long-term memory are key to the undisputed performance of biological neural networks. Here, we report on sub-µm-sized artificial synaptic weights exploiting a combination of a ferroelectric space charge effect and oxidation state modulation in the oxide channel of a ferroelectric field effect transistor. They lead to a quasi-continuous resistance tuning of the synapse by a factor of $60$ and a fine-grained weight update of more than $200$ resistance values. We leverage a fast, saturating ferroelectric effect and a slow, ionic drift and diffusion process to engineer a multi-timescale artificial synapse. Our device demonstrates an endurance of more than ${10}^{10}$ cycles, a ferroelectric retention of more than $10$ years, and various types of volatility behavior on distinct timescales, making it well suited for neuromorphic and cognitive computing.

## Abstract (web summary)

Brain-inspired neuromorphic computing is a key technology for processing an ever-growing amount of data. Here, an artificial synapse with dual resistance modulation mechanisms is demonstrated, achieving a dynamic range of 60, an endurance exceeding 10^10^ cycles, and more than 10 years of retention.

## Introduction

The amount of data created during the last thirty years (~320 ZB) compares with what will be created during the next three years (~364 ZB projected for 2022–2024), a trend also accelerated by the covid-19 pandemic^1–3^. A considerable amount will be created by the rapidly growing Internet of Things (IoT)^4^, which connects the physical world and computing entities. The development of sensors and actuators that connect to the internet, comes in pair with the emergence of Artificial Neural Networks (ANNs) for data processing. The conventional von-Neumann architecture cannot sustain such evolution, because of the energy and performance bottleneck coming from the massive data movement between the physically separated memory and processing units. Novel processing architectures, device technologies, and computational paradigms have therefore recently emerged. In-memory computing^5^ co-locates memory and processing, eliminating inefficient data movement, which is especially beneficial for ANN training by data-intense machine-learning algorithms. In addition to ANNs, computing systems like Spiking Neural Networks (SNNs), which mimic the type of information processing in the human brain, promise low-power computation and dynamic learning in the context of complex data^6^. A major challenge for SNNs remains in supporting operations under a wide range of effective timescales^7^. This requirement arises from the need to adapt the computation to the input timescale in real-time online applications (e.g., real-world sensory signals) and because multi-timescales are inherent in spiking neurodynamics (e.g., neural activation decay, combination of short- and long-term plasticity mechanisms^7–9^). To emulate the complex and multi-timescale plasticity processes of biological synapses^7^, physical effects acting at different timescales must be orchestrated in an artificial synapse^9^. Being able to tune the timescale (tunable volatility) in SNNs would bring the ability to mimic many of the basic processing and storage operations of the mammalian brain^10^ and facilitate reservoir computing or unsupervised learning^11^.

In ANNs and SNNs, analog non-volatile resistive memory elements, so-called memristors, are used to emulate synaptic functionality, to locally store network parameters^12,13^, and to serve as analog computing element^14^. Lately, multi-terminal memristive devices have gained a lot of attention due to the ability of using extra terminals to tune their switching dynamics (volatility, plasticity timescales), opening opportunities to implement advanced bio-inspired learning rules. For example, reward-modulated spike-timing-dependent plasticity was demonstrated in a 4-terminal cell based on ferroelectric P(VDFTrFE)^15^. The concept of multi-terminal can also be extended to combining electrical and light spikes to control the synapse plasticity^16^. Hafnia-based technologies^17–19^ are CMOS-compatible; the low-power and multi-state nature of hafnia-based Ferroelectric Field-Effect Transistors (FeFETs) makes them a viable candidate for the development of a multi-timescale synaptic element in neuromorphic circuits. FeFETs^20–22^ have the advantage over two-terminal devices of separating the read and write path^23^. On one hand, this permits to write to a high-impedance gate with low-power. On the other hand, the reading current flows through the channel that can be engineered to be ohmic^24^.

Scaled FeFETs nevertheless suffer from a finite number of conductance levels^21^ due to the discrete number of ferroelectric domains available in each device. Integrating the FeFETs in the Back-End-Of-Line (BEOL) leaves more area for the control electronics while at the same time it enables analog weight updates through relaxed size constraints^24–26^.

In this work we present a BEOL-compatible, back-gated FeFET utilizing a 10 nm thick HfZrO_4_ (HZO) ferroelectric gate dielectric and a 4 nm tungsten oxide (WO_x_) thin-film channel. The screening of the ferroelectric polarization charges of HZO results in an accumulation or depletion of electrons in the WO_x_ channel, effectively changing its resistivity. Beyond the absence of interfacial dielectric layers (which limit the performance of FeFETs with doped-Si channels^27^), oxide channels are of interest for the intrinsic presence of slow ionic drift and diffusion mechanisms, and hence are viable candidates for a slow plasticity timescale effect. WO_x_ was chosen due to its relatively mobile oxygen-ions and thereby tunable conductivity as a function of oxygen content^28,29^. Furthermore, the junction less contacts to the WO_x_ permit a ohmic conduction between source and drain^24^.

We engineered our device by using a channel with the appropriate stoichiometry and by having it in direct contact with HZO. In the first section, we capture how these measures facilitate a resistance modulation of the channel when the device is programmed with different timescales: on one hand the resistance modulation is driven by fast electronic screening of the HZO polarization charges, and on the other hand by the slow ionic drift and diffusion process of oxygen in the WO_x_. In the second section, we characterize the synaptic weight and provide an evaluation of the proposed technology’s performance when implemented in an ANN.

## Results and discussion

### Artificial synaptic weight structure

**Fig. 1:**
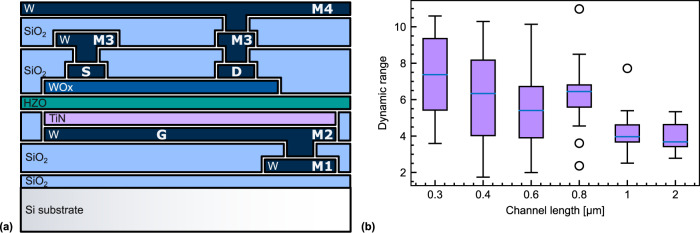
Structural data of the FeFET. **a** Schematic illustration of the FeFET, indicating a source (S), a drain (D), a gate (G), a WO_x_ channel, a ferroelectric HZO gate dielectric, G access metal line (M1, M2), S access metal line (M3), and D access metal line (M4). **b** Channel length effect: The dynamic range of $120$ devices with a channel width of 600 nm and a varying channel length from 300 nm to 2 μm. Write pulses with an amplitude (${V}_{{{\rm{w}}}}$) from −6 *V* to 6 *V* and width (${t}_{{{\rm{w}}}}$) of 500 μ*s* were applied. The same data for (*t*_w_ = 500 ms are available in Supplementary Fig. 6a. The boxes extend from the lower to upper quartile values of the data, with a line at the median. The whiskers extend from the box to show the range of the data. Flier points are those past the end of the whiskers.

We developed a back-gated FeFET with two metal lines (M1, M2) for the Gate (G) and two (M3, M4) for the Source (S) and Drain (D) to establish a three-terminal device configuration. The FeFET gate stack is made of a TiN contact and a 10 nm HZO ferroelectric gate dielectric. The thin-film channel consists of a 4 nm WO_x_ layer on top of the HZO. The S and D contacts are made of Pt/W and the passivation layer above the WO_x_ consists of an Al_2_O_3_ and a SiO_2_ layer. In our design, the gate fully overlaps with S and D to avoid large topographic steps beneath the channel. The device schematic is depicted in Fig. 1a. In this work, single, stand-alone devices are studied. In view of their integration in passive pseudo-crossbar circuits, the geometry of the channel is designed to optimize the channel resistance (we refer to Supplementary Note 1 where the pseudo-crossbar operation is further discussed). For the integration in active pseudo-crossbar circuits (as proposed for example by Jerry et al.^20^), the devices would be embedded in at least four metal levels. Therefore, within our stand-alone device processing scheme, we include these challenges inherent to the complexity of the active crossbar realization.

The structural analysis of our device material stack is described in Supplementary Note 2. Grazing-Incidence X-Ray Diffraction GIXRD scans confirm the absence of the monoclinic phase, the peak at $30.7^\circ$ corresponds to the ferroelectric orthorhombic phase of HZO^30^. Bright-field Scanning Transmission Electron Microscopy (BF-STEM) analysis confirms the expected layer thickness. Energy-dispersive X-ray spectroscopy reveals the diffusion of Al into the WO_x_ layer, a metal expected to promote the reduction of WO_x_^31^.

In addition to the ferroelectric field-effect, this work exploits the high mobility of oxygen in WO_x_, as observed during reduction anneals^32^ and in WO_x_ nanowires^33^. More generally, WO_x_ was chosen as a channel for its electron-doping ability through the removal of oxygen^34,35^. In this work, the as-deposited WO_x_ was close to a stoichiometry of $x=3$ (insulating). We then leverage the mobility of oxygen: during the deposition of the passivation layers (Al_2_O_3_ and SiO_2_), the WO_x_ was slightly reduced (consistently with the Al diffusion found earlier). The resulting mobility and carrier concentration are suitable for a large channel conductance modulation by the polarization charges via Coulomb coupling^29^: the screening of the HZO polarization charges in the WO_x_ occurs over a distance comparable to the channel thickness^24^. A 2D time-dependent Ginzburg-Landau model of the HZO/WO_x_ FeFET^36^ provided guidelines for the optimization of the channel thickness and stoichiometry. Moreover, the resulting channel resistances in the MΩ to GΩ range are well suited for energy-efficient and scalable crossbar array operation^37^.

The capacitance, resistance, and polarization characterization of the gate stack are found in the Supplementary Notes 3 and 4.

### Switching components and timescales

Potentiation (depression) cycles on $120$ devices with constant channel width (*W*_ch_ = 600 nm) and a channel length varying from *L*_ch_ = 300 nm to 2 μm were measured by applying write pulses with an amplitude (${V}_{{{{{{\rm{w}}}}}}}$) up to 6 V (−6 V) and a width (${t}_{{{{{{\rm{w}}}}}}}$) of 500 μs. In this work, the device is programmed symmetrically, i.e., by applying ${V}_{{{{{{\rm{w}}}}}}}$ on the gate while grounding both the S and D contacts. The dynamic range (DR) is defined as the ratio of the source-to-drain resistance (${R}_{{{{{{\rm{SD}}}}}}}$) in the high resistive state (HRS) to the low resistive state (LRS). First, the influence of the channel length on the DR is examined by performing a statistical analysis on $120$ devices to average out variations due to local fabrication inhomogeneities (see further discussion in section “Characteristics for ANNs”). The average DR and the distribution are represented as a function of ${L}_{{{{{{\rm{ch}}}}}}}$ in Fig. 1b. A detailed analysis of the DR dependence on ${L}_{{{{{{\rm{ch}}}}}}}$ is found in the Supplementary Note 5. We found that the two components of the source-to-drain resistance ${R}_{{{{{{\rm{SD}}}}}}}={R}_{{{{{{\rm{ch}}}}}}}+{2R}_{{{{{{\rm{c}}}}}}}$, the channel resistance ${R}_{{{{{{\rm{ch}}}}}}}$ and the contact resistance ${R}_{{{{{{\rm{c}}}}}}}$, are modulated to a different extend by the write pulses. While ${R}_{{{{{{\rm{c}}}}}}}$ displays a modulation of ${{DR}}_{{R}_{{{{{{\rm{c}}}}}}}}=8.4$, the dynamic range of the channel ${{DR}}_{{R}_{{{{{{\rm{Ch}}}}}}}}=2.8$ is three times less. Consequently, the total DR in Fig. 1b, that includes both contributions, decreases for longer channels as the relative contribution of ${{DR}}_{{R}_{{{{{{\rm{Ch}}}}}}}}$ increases. Moreover, an overall increase of the DR by a factor of ~2.8 was observed by increasing ${t}_{{{{{{\rm{w}}}}}}}$ to 500 ms, a first indication of another ${R}_{{{{{{\rm{SD}}}}}}}$ modulation than caused by the polarization switching at short ${t}_{{{{{{\rm{w}}}}}}}$ (500 μ*s*).

**Fig. 2:**
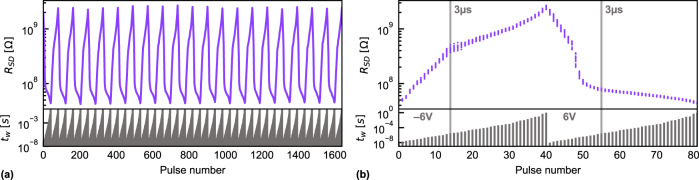
Potentiation and depression cycles with a constant pulse amplitude scheme. The write pulse width ${t}_{{{\rm{w}}}}$ was modulated from 10 ns to 1 s while keeping the write pulse amplitude ${V}_{{{\rm{w}}}}$ constant at 6 *V* (−6 *V*) for the potentiation (depression) on a FeFET with *L* = 400 nm and *W* = 1 μm. **a** Source-drain resistance as a function of the pulse number for $20$ consecutive cycles. On the bottom, the corresponding duration ${t}_{{{\rm{w}}}}$ of each pulse is provided. **b** Superposition of the data points shown in **a**) and represented as a function of the pulse number in each cycle, to visualize the small cycle-to-cycle variations and potentiation and depression shape.

The influence of ${t}_{{{\rm{w}}}}$ on the dynamic range was further analyzed by performing a sequence of 20 constant amplitude potentiation (${V}_{{{{{{\rm{w}}}}}}}=6\ V$) and depression (${V}_{{{{{{\rm{w}}}}}}}=-6\ {V}$) cycles with changing ${t}_{{{{{{\rm{w}}}}}}}$ from 10 ns to 1 s (Fig. 2a). In Fig. 2b, the same 20 potentiation/depression cycles are superposed, to highlight the low cycle-to-cycle variability and better visualize the potentiation and depression shape. A dynamic range of almost 60 was reached (average of 20 cycles), a considerable increase as compared to the state-of-the-art oxide-channel FeFETs^24,25^. The ${R}_{{{{{{\rm{SD}}}}}}}$ shows a strong dependence on ${t}_{{{{{{\rm{w}}}}}}}$: there are two different regimes for both the potentiation and depression. For short pulses (*t*_w_ > 3 μs, ${V}_{{{{{{\rm{w}}}}}}}=-6\ {V}$) a steep depression is observed, followed by a less steep change (*t*_w_ > 3 μs, ${V}_{{{{{{\rm{w}}}}}}}=-6\ {V}$) that does not saturate up to the maximum ${t}_{{{{{{\rm{w}}}}}}}$ of 1 s. In the long pulse regime, cumulative switching is observed: repeating the same pulse (e.g., *t*_w_ = 100 ms, ${V}_{{{{{{\rm{w}}}}}}}=-6\ {V}$) 100 times increased R_SD_ after the first 10 cycles still by a factor 2 (Supplementary Note 6). The absence of a saturation and a change of slope indicates that the resistance modulation for *t*_w_ > 3 μs is based on an additional and slower physical process with respect to the previous regime (*t*_w_ > 3 μs): the first regime (*t*_w_ > 3 μs) is attributed to the ferroelectric switching as it is known to occur at very fast timescales^38^. In the second regime (*t*_w_ > 3 μs), the energy of each pulse was potentially large enough to additionally enable oxygen migration^33,39^ between HZO and WO_x_. The oxidation or reduction of the WO_x_ channel in turn adds to the modulation of ${R}_{{{{{{\rm{SD}}}}}}}$.

The underlying conduction mechanisms of the two regimes are discussed next. Several electrode-limited and bulk-limited conduction mechanisms depend on temperature in different ways^40^. Temperature-dependent ${I}_{{{{{{\rm{DS}}}}}}}-{V}_{{{{{{\rm{DS}}}}}}}$ measurements of the channel were performed (20° C–60 °C) after programming the device in the LRS (*V*_w_ = 6 *V*) and in the HRS (${V}_{{{{{{\rm{w}}}}}}}=-6\ {V}$). From Fig. 2b, we observe that fast, ferroelectric effects occur already from pulse widths of 100 ns. The onset of the change of regime is observed for pulses of 3 μs. To reveal that there are indeed two different mechanisms to modulate the resistance, the effect of the temperature on the DR was investigated at different timescales. Write pulses of *t*_w_ = 500 μs (where the ferroelectric effect is fully saturated and oxygen migration just starts) and longer write pulse trains of 90 · *t*_w_ = 100 ms (to enhance the oxygen migration) were applied.

No difference in the conduction mechanism between the two timescales was observed, as discussed in Supplementary Note 7. Both the LRS and HRS show a linear I_D_-V_DS_ characteristic and are best fitted with the ohmic conduction model^41^.

**Fig. 3:**
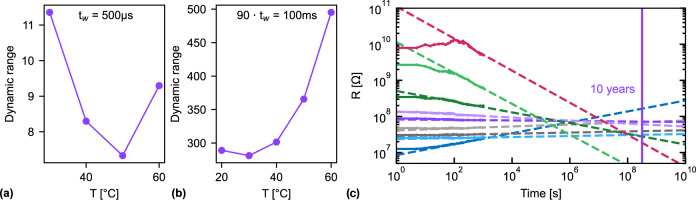
Temperature-dependent measurements. Dynamic range modulation with temperature for **a** *t*_w_ = 500 μs and **b** longer write pulse trains of 90 · *t*_w_ = 100 ms. **c** Retention measurements at elevated temperature (85 °C) for long set pulses *t*_w_ = 500 ms.

In contrast, comparing the DR as a function of temperature for the two timescales (Fig. 3a, b) displays a clear difference. For the pulses where the ferroelectric effect dominates (*t*_w_ = 500 μs), the DR decreases with increasing temperature. This moderate effect can be attributed to a phase transition from the orthorhombic ferroelectric phase of HZO to its tetragonal, anti-ferroelectric phase at elevated temperatures, as observed in ZrO_2_^42^ and Si:HfO_2_^43^. From 50° C–60 °C, an increase in the dynamic range is observed, indicating an additional mechanism that modulates the resistance becoming dominant. This is explained by the chosen pulse width of *t*_w_ = 500 μs that ensures saturation of the polarization but is also large enough for the second effect to become noticeable (predicted by Fig. 2b for pulses above 3 µs). In contrast, for the slower timescale, an increase in the dynamic range with temperature is observed in the whole temperature range and is one to two orders of magnitude larger. From 50° C–60 °C, the relative increase in the dynamic range is the highest. The temperature range was limited to 20° C–60 °C since above, irreversible changes of the gate were observed for some devices when applying a bias of 6 V. These observations are consistent with increased oxygen-ion mobility at elevated temperatures, which facilitates the channels oxidation or reduction. In this scenario, the observed increase of the DR for 500 μs pulses above 50° C suggests a dual dependency of the oxygen-ion mobility on the temperature and on the pulse duration. The different types of behavior with temperature confirm the presence of two effects modulating our channel resistance. A schematic diagram illustrating the two effects can be found in Supplementary Fig. 10.

Retention measurements at elevated temperatures (85 °C) and with long write pulses (*t*_w_ = 500 ms display a DR of almost three orders of magnitude, a direct consequence of the aforementioned increased oxygen-ion mobility (Fig. 3c). The larger the resistance change obtained within this retention study, the faster (hours to minutes) it drifted back. The improved retention of the intermediate states compared to the extreme states rules out the effect of a depolarization field, as observed by Muller et al. in TiN/SiHfO_2_/TiN capacitors^44^. Furthermore, the observation that both the LRS and HRS relax towards intermediate resistances confirm that the dominant mechanism is not a depolarization field due to a limited number of carriers in the WO_x_. Therefore, a resistance modulation by displacing oxygen-ions will eventually relax back to equilibrium by ion-diffusion processes, introducing the possibility to tune the plasticity timescale by controlling the oxygen-ion mobility. The more consecutive pulses are applied, the larger the resistance change and the longer the relaxation back to a stable long-term state. This characteristic can be used to implement short-term plasticity^45^. In dedicated neuromorphic hardware, the above-mentioned relaxation occurs on long timescales (seconds, minutes, hours): this should then also hold for the stimuli or events processed by the network, as for example in the application field of health monitoring or autonomous driving. At shorter timescales, our ferroelectric synaptic functionalities ascribed to the existence of a threshold voltage for domain switching (coercive field), makes our devices good candidates for implementation in spiking neural networks. For example, spike-timing-dependent plasticity (STDP) was reported in perovskite^46^ and hafnia^47^ based thin films. Finally, the gradual and field-driven switching of the ferroelectric domains is well suited for implementing voltage-based STDP models^48–50^. In recent work, Garg et al.^51^ showed that such unsupervised, voltage-dependent-plasticity learning rules outperforms STDP in classification tasks.

Building on this finding of multiple timescales and physical locations where the ${R}_{{{{{{\rm{SD}}}}}}}$ modulation occurs, we envision adding a 4th electrode with a non-ferroelectric gate on top of the WO_x_ dielectric to further oxidize or reduce the channel without affecting the ferroelectric state. This additional terminal provides a volatile resistance component to tune the switching dynamics, similarly to^52^. Having such an additional volatile component permits the implementation of a wide range of neuromorphic engineering paradigms^53^.

### Characteristics for ANNs

**Fig. 4:**
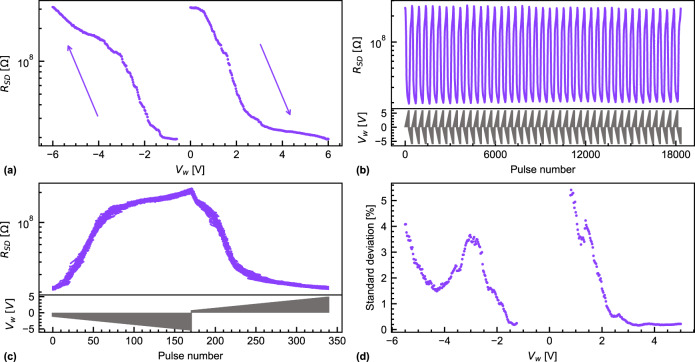
Potentiation and depression cycles with a constant pulse width scheme. The FeFET has a channel length of 300 nm and a channel width of 2 μm: **a** one potentiation (0 V to 6 V) and depression (−0.6 V to −6 V) cycle with a constant *t*_w_ = 500 μs showing quasi-continuous channel resistance states (dep: $241$ states, pot: 217). **b** $40$ sub-range potentiation (0.6 V to 5 V, $171$ levels) and depression (1.25 V to −5.5 V, $169$ levels) cycles. Channel resistance R_SD_ as a function of pulse number (top) and the corresponding ${V}_{w}$ (bottom) are reported. **c** Superposition of all $40$ cycles from (**b**) to visualize the cycle-to-cycle variability. **d** Standard deviation of a channel resistance state normalized by the resistance window (HRS-LRS) for each ${V}_{{{\rm{w}}}}$.

In contrast to SNNs, where a tunable plasticity is desired, the efficient operation of ANNs on analog memristive crossbar arrays requires artificial synapses that behave as long-term memory with long data retention and low variability. Figure 4a shows a single potentiation and (depression) cycle of ${R}_{{{{{{\rm{SD}}}}}}}$ by increasing (decreasing) ${V}_{{{{{{\rm{w}}}}}}}$ from 0 V to 6 V (−0.6 V to −6 V) in 25 mV steps. The programming and ${R}_{{{{{{\rm{SD}}}}}}}$ measurement protocols are described in the section “Methods, Electrical characterization”; for a sampling bias of 200 mV, the conduction in the channel is Ohmic. The pulse duration *t*_w_ was kept constant at 500 μs. The 241 (217) steps for the potentiation (depression) exhibit a quasi-continuous resistance range with a monotonic change of the resistance and a DR of $16$. The Cycle-to-Cycle Variability (CtCV) was analyzed by performing $40$ sub-range cycles of depression (−0.6 V to −5 V) and potentiation (−1.25 *V* to −5.5 *V*) as shown in Fig. 4b. The corresponding ${V}_{{{{{{\rm{w}}}}}}}$ to each pulse is given at the bottom. Figure 4c is a superposition of all $40$ cycles to help visualize the CtCV. By reducing the ${V}_{{{{{{\rm{w}}}}}}}$ range, the DR decreases to $10.4$ on average. Figure 4d is a visualization of the CtCV (standard deviation) as a percentage of the channel resistance range (HRS-LRS). The CtCV does not exceed $6\, \%$ and is as low as $1.9\, \%$ on average.

To study the device-to-device variability, $20$ identical FeFETs were measured and the normalized standard deviation of the HRS ($39 \%$), LRS ($39 \%$), and DR ($28 \%$) was extracted. The device-to-device variability can be explained by process variations across the sample. The polycrystalline nature of HZO results in different ferroelectric properties from device to device^19^. Also, the WO_x_ conductivity is rather sensitive to its oxygen content and can be reduced at elevated temperatures (>250 °C) by other oxides or nitrides interfacing it, such as SiO_2_, Al_2_O_3_, or SiN. Hence, local temperature differences during processing can cause different local reduction states of the WO_x_.

**Fig. 5:**
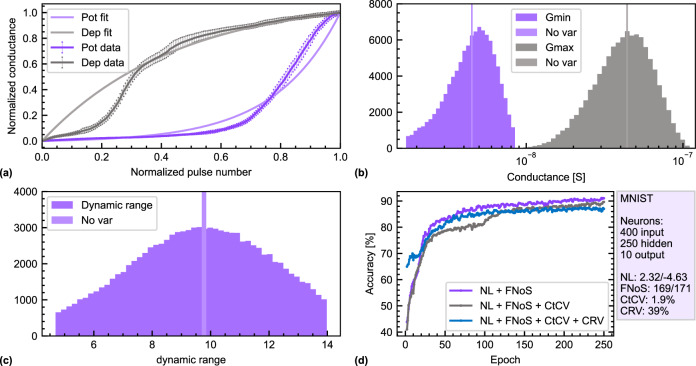
Online learning performed with MLP+ NeuroSimV3.0. **a** Exponential fit of the potentiation and depression curves to extract the corresponding non-linearity parameter. **b**, **c** Histogram of the HRS, LRS, and DR after taking the device-to-device variability into account. **d** MNIST classification performance of our FeFETs with different degrees of non-idealities included: non-linearity factors and finite number of steps (purple), + cycle to cycle variation (gray), and + conductance range variation (blue).

The performance of our FeFETs as artificial synapses in a crossbar array was investigated by using the MLP+ NeuroSimV3.0^54^ framework. The on-line learning accuracy of a pseudo-crossbar array of $400$ input, $250$ hidden, and $10$ output neurons trained on the MNIST database was simulated by using the aforementioned values. The non-linearity (NL) parameters were extracted by fitting the potentiation and depression curves according to ref. ^54^ (Fig. 5a). They are $2.32$ and $-4.63$ for the potentiation and depression, respectively. Moreover, Fig. 4a shows that operating the FeFET in a subloop regime (±3V) would improve the linearity even further, but at the cost of a smaller dynamic range. Here, the MLP+ NeuroSimV3.0 code was slightly adapted (Supplementary information Note 8) to apply a random Conductance Range Variation (CRV) to every device in the network, which takes into account our device-to-device variability. The spread of the HRS, LRS, and DR around the average (No var) are depicted in Fig. 5b, c. Figure 5d reports the learning accuracy on the MNIST database. By only considering the NL parameters and the Finite Number-of-States (FNoS) as a non-ideality, an excellent performance of $92 \%$ recognition accuracy is achieved. This metrics show that the proposed technology compares to state-of-the-art FeFET evaluated with the same platform (Supplementary Table 3). By further introducing the CtCV to the simulation, the performance remains as high as $89 \%$ and with the HRS, LRS, and DR spread included, still 88% is reached.

**Fig. 6:**
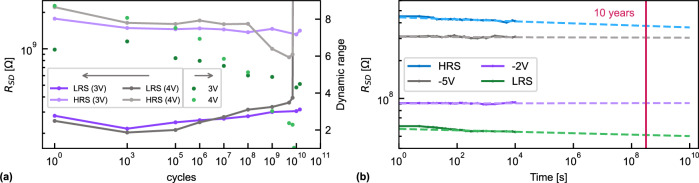
Endurance and retention. **a** Endurance of a FeFET with *L* = 800 nm and *W* = 600 nm. Triangular pulses with a frequency of 100 kHz were applied up to 10^10^ cycles. The amplitude of the pulses was ±3 V and ±4 V. The evolution of the HRS and LRS (left axis) and the corresponding dynamic range (right axis) are shown. **b** Retention measurements at room temperature for a FeFET with *L* = 300 nm and *W* = 2 μm showing a good retention of >10 years for the four programmed states. Only the HRS has a small drift. The solid lines are the experimental data and the dashed lines are linear extrapolations in the log-log scale.

We then perform endurance and retention tests to evaluate the benefit of using a metallic oxide channel, compared to state-of-the-art FeFET on Si, for which is typically reported an endurance of 10^6^ ^19^ cycles, although lately up to 10^8^ ^55^ cycles were reported. The endurance of a FeFET with *L* = 800 nm and *W* = 600 nm is shown in Fig. 6a. Cycling pulses of ±3 V and ±4 V with a frequency of 100 KHz were applied to the gate, while the source and drain were grounded. When switching at ±3 V we observed a small continuous decay of the DR to ~70% of its initial value after ${10}^{10}$ cycles, but no failure could be identified. To the best of our knowledge this is the best endurance reported on hafnia-based FeFETs. Especially when compared with Si-based channel FeFETs this is a major improvement. Increasing the cycling voltage to $\pm \,4{V}$ (on another device with the same dimensions) accelerated the fatigue and the device failed after $8 \bullet {10}^{9}$ cycles. Cycling at even higher fields was not tested as online learning happens in small changes and not by constantly switching between the extreme states.

With a channel resistance between 20 MΩ and 2 GΩ (depending on the geometry) and a read voltage of 100 mV, between 5 pW and 500 pW are dissipated during a read operation (${P}_{{{{{{\rm{read}}}}}}}={V}_{{{{{{\rm{SD}}}}}}}^{2}/{R}_{{{{{{\rm{SD}}}}}}}$). The write operation of a single device (*t*_w_ = 500 μs) to the high gate impedance (Supplementary Fig. 4b) has a lower energy consumption, between 1.7 fJ and 1.2 pJ for ${V}_{{{{{{\rm{w}}}}}}}$ of $1{V}$ and $6{V}$, respectively (${E}_{{{\rm{write}}}}={{{{{{\rm{t}}}}}}}_{{{{{{\rm{w}}}}}}}\bullet {V}_{{{{{{\rm{w}}}}}}}^{2}/{R}_{{{{{{\rm{G}}}}}}}$). The low-power operation, long endurance, and promising MNIST classification performance make this a viable candidate for large crossbar implementations for ANN training.

Retention measurements are depicted in Fig. 6b. By fitting a linear regression in the log-log scale, no drift is observed for the intermediate states. Both, the LRS and HRS display a small drift towards lower values. Extrapolating the fit to $10$ years yields a change of about $11 \%$ and $15 \%$, respectively. This excellent retention time confirms the advantage of using metal-oxide thin films over Si as channels as there is no back-switching of the ferroelectric domains due to charge trapping at the oxide interlayer formed between Si and the ferroelectric^56^. This low drift opens the path to inference and memory applications for the FeFET devices presented in this study.

## Conclusion

We demonstrated a scaled (sub-µm) BEOL-compatible FeFET artificial synapse with an amorphous WO_*x* < 3_ channel. The device concept was engineered to leverage two controllable resistance modulation mechanisms activated on two different write pulse timescales: a fast ferroelectric field effect (*t*_w_ < 3 μs) and an oxidation/reduction of the channel by oxygen movement at a slower timescale (*t*_w_ > 3 μs). Key enablers were the control of the channel oxidation state and of its thickness down to 4 nm, as well as having the channel in direct contact with the ferroelectric HZO gate without the formation of a spurious interlayer.

The dual nature of the resistance modulation mechanisms was derived from the write time-dependent dynamics and from the two different potentiation and depression slopes. The temperature-dependent current measurements showed opposite dynamic range trends for the two timescales, confirming that the resistance changes originate from two different mechanisms. Moreover, the temperature-dependent retention measurements highlighted the role of the oxygen drift across the layers in the slow regime (*t*_w_ > 3 μs).

With this extra option to extend the dynamic range, our scaled FeFETs have a dynamic HRS/LRS ratio at room temperature that is 30 times larger than the state-of-the art^24^. The plasticity of our synapse shows a different response over multiple timescales, making our FeFETs interesting candidates for neuromorphic engineering. The ohmic nature and a resistance of our WO_x_ channel in the MΩ regime are excellent features for precise and low-power readout operations. The extremely fine-grained, quasi-continuous monotonic resistance changes with more than 200 steps between the LRS and HRS, together with an excellent cycle-to-cycle variability led to a good MNIST classification accuracy of $88 \%$ with the NeuroSim framework. The endurance was extended to >10^10^ cycles and an excellent retention of >10 years was obtained with only little dynamic range loss. Therefore, our FeFET technology is not only promising for online learning but also for in-memory computing and neural network inference applications. A 4th electrode acting as top gate could introduce a volatile component, a desired property for bio-inspired neuromorphic engineering paradigms.

## Methods

### Sample preparation

Our FeFET is a back-gated device to allow the crystallization of the ferroelectric HZO gate dielectric before the deposition of the thin-film WO_x_ channel. First, 500 nm SiO_2_ were formed by thermally oxidizing the Si substrate. A 100 nm thick W layer was deposited by sputtering and consecutively etched in a Reactive Ion Etcher (RIE) with an SF_6_ plasma to form the first gate metal level (M1). Then, a 100 nm thick SiO_2_ passivation was deposited by Plasma-Enhanced Chemical Vapor Deposition (PECVD) at 300 °C and vias to M1 were etched using an RIE with a CHF_3_/O_2_ plasma. M2 was deposited as M1 and continued by a 10 nm TiN layer deposited using a tetrakis- (dimethylamino)titanium (TDMAT) precursor and N_2_/H_2_ plasma in an Oxford Instruments plasma-enhanced atomic layer deposition (PEALD) system. To avoid large topographies, 600 nm of SiO_2_ were deposited on top of M2 and then removed by chemical mechanical polishing (CMP) until only a thin layer of SiO_2_ was left above M2. This effectively removed the topography introduced by M1 and M2 (Supplementary Fig. 3d). The last few nm of SiO_2_ were then etched by RIE to expose the TiN on top of M2. An ~10 nm thick layer of HZO was grown in a process using alternating cycles of tetrakis-(ethylmethylamino)-hafnium (TEMAH) and bis-(methyl-η5-cyclopentadienyl)-methoxymethyl-zirconium (ZrCMMM) at 300 °C and then capped by 30 nm of W deposited by sputtering. The crystallization of the HZO was then performed by a millisecond flash lamp anneal (ms-FLA)^30^ where the sample was heated to 375 °C. After the crystallization the W was removed by a wet etch in H_2_O_2_ at 50 °C. 4 nm of WO_x_ was then deposited using a bis-(tert-butylimino)-bis-(dimethylamino)-tungsten precursor and an oxygen plasma at 375 °C in a PEALD system. To fully oxidize the WO_x_, it was annealed in a rapid thermal annealer at 350 °C with 50 sccm O_2_ for $6\,\min$. After the structuring of the WO_x_ by an SF_6_ plasma in the RIE, source (S) and drain (D) contacts (Pt/W) were deposited on top of the WO_x_ channel by lift-off. Finally, the structures were passivated by 100 nm of SiO_2_ and two metal levels (M3, M4) were fabricated as M1 and M2.

### Structural characterization

Grazing-Incidence X-Ray Diffraction (GIXRD) measurements were executed on a Bruker D8 Discover diffractometer equipped with a copper rotating anode generator. Cross-sectional cuts and lamellas for Scanning Transmission Electron Microscope (STEM) analyses were prepared by Focused Ion Beam (FIB) using an FEI Helios NanoLab 450 S. STEM analysis was carried out on a double spherical aberration-corrected JEOL JEM-ARM200F microscope. Bright-Field STEM (BF-STEM) images were acquired at 200 kV, and energy-dispersive X-ray spectroscopy (EDS) line profiles were performed using a liquid-nitrogen-free silicon drift detector.

### Electrical characterization

The electrical characterization was performed using a probestation in atmosphere. For the temperature-dependent measurements, the stage was heated and the temperature controlled by a thermocouple. Otherwise, the measurements were performed at room temperature. Potentiation and depression, resistance, retention, endurance, and capacitance measurements were performed on an Agilent B1500A with a B1530A waveform generator/fast measurement unit and a B1520A multi-frequency capacitance measurement unit. Write pulses were generated by a remote sense unit module close to the probe and applied to the gate while source and drain were grounded. To read the channel resistance, four sampling points at *V*_DS_ = 200 mV and *V*_DS_ = −200 mV were taken with the source measurement unit at the source while the drain was grounded and the gate was floating. PUND measurements on MFSM capacitors were performed on a TF analyzer 2000 from aixACCT. The PUND signal of ±5 V at 1 kHz was applied to the W/TiN contact (equivalent of the gate in a FeFET) of the W/TiN/HZO/WO_x_/W capacitor, while the other side was grounded. Before the PUND measurement, devices were woken up by ${10}^{5}$ fully switching cycles with an amplitude of ±4 V.

## Supplementary information

Supplementary Information

## Data Availability

The data that support the findings of this study have been included in the manuscript and supplementary information. Any additional data are available from the corresponding author upon reasonable request.
