# Supplementary material for: A multi-timescale synaptic weight based on ferroelectric hafnium zirconium oxide
Source: Commun Mater. 2023 Feb 17;4(1):14. doi: 10.1038/s43246-023-00342-x (PMC9936949; doi:10.1038/s43246-023-00342-x)
Supplement: Supplementary file 1 — Supplementary Information [file 43246_2023_342_MOESM1_ESM.pdf]

A multi-timescale synaptic weight based on ferroelectric hafnium zirconium oxide

Mattia Halter[1,2,3]*, Laura Bégon-Lours[1], Marilyne Sousa[1], Youri Popoff[1,2], Ute Drechsler[1], Valeria Bragaglia[1], Bert Jan Offrein[1]

[1]IBM Research Europe - Zurich Research Laboratory, CH-8803 Rüschlikon, Switzerland,

[2]ETH Zurich - Integrated Systems Laboratory, CH-8092 Zurich, Switzerland,

[3]Current Address: Lumiphase AG, CH-8712 Stäfa, Switzerland

*mattia@lumiphase.com

Supplementary Information

Supplementary Note 1. Passive pseudo-crossbar operation

In a passive crossbar (**Supplementary Figure 1**), it is beneficial to have a gate resistance that compares to the channel resistance. In this schematic, the gate of device 11 is addressed: A parallel path (yellow dots) exists. If the channel resistances $r12$, $r22$ and $r21$ are small compared to the resistance of the gate $R12$, the gate of device 12 also sees a difference of potential equal to VG.

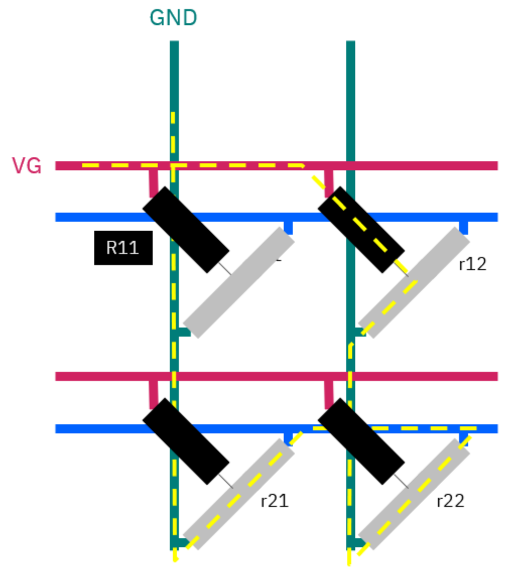

Supplementary Figure 1: Schematics of a 2x2 passive pseudo-crossbar. The device 11 is addressed. The voltage drops through a parallel path, where the gate of the device 12 is in series with the channels of devices 21, 22 and 12.

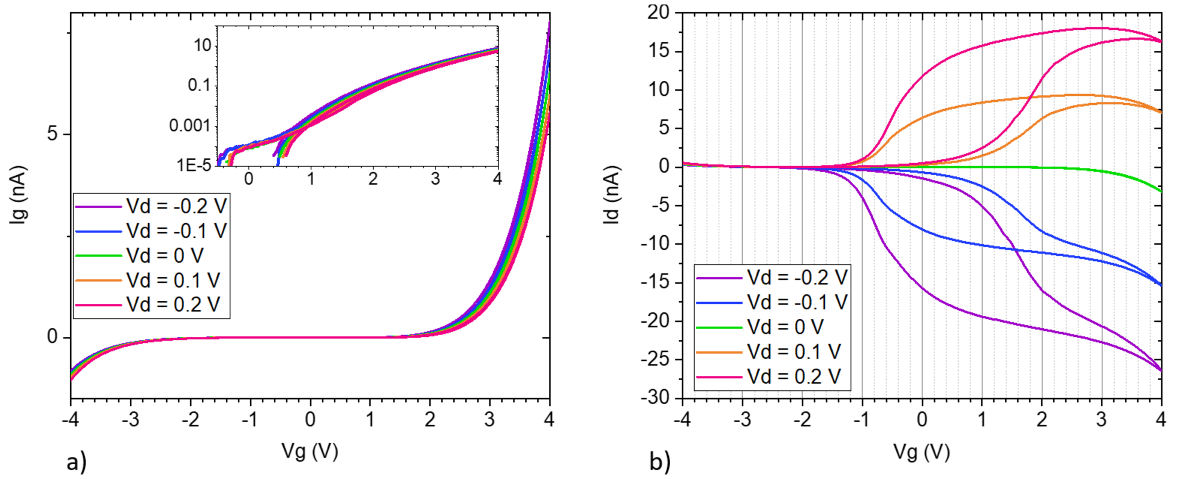

Supplementary Figure 2: Gate leakage and Id-Vg characteristics for a stand-alone FeFET. (a) shows the gate leakage and **(b)** the $I_d - V_g$ characteristics of a FeFET with a channel length of 500 nm and a channel width of 500 nm.

Supplementary Note 2. Structural analysis

Grazing-Incidence X-Ray Diffraction (GIXRD) scan, taken after the crystallization of HZO and the extra WO_x oxidation in a Rapid Thermal Annealer (RTA), is shown in **Supplementary Figure 3(a)** and confirms the ferroelectric orthorhombic phase of HZO. No monoclinic phase is observed, confirming the effectiveness of our Back-End-Of-Line (BEOL) compatible crystallization[1]. Further, the analysis indicates that the WO_x stayed amorphous during the RTA oxidation step as the characteristic peaks are missing. For comparison, a GIXRD scan of a crystallized WO_x (ICSD86144)[2] is shown in **Supplementary Figure 3 (a)**.

Bright-Field Scanning Transmission Electron Microscopy (BF-STEM) analysis displays the expected layer thicknesses and an amorphous WO_x (**Supplementary Figure 3 (b)**). The rough topography of the W gate, originating from its columnar growth (**Supplementary Figure 3 (d)**), results in a projection effect (overlapping of measured elements) at layer boundaries as seen in the Energy-Dispersive X-ray Spectroscopy (EDS) line profile in **Figure 1(b)**. Although the projection effect creates some uncertainty in the interpretation of the EDS data, two observations can be made: First, at the HZO/TiN interface a TiON layer was formed, possibly during the HZO growth by Atomic Layer Deposition (ALD). Second, Al diffusion into the WO_x is measured (more pronounced than the artificial mixing by the projection effect). This is interesting as Al is expected to promote the reduction of WO_x[3]. (By reducing WO_3 to WO_{3-x}, oxygen vacancies not only donate delocalized electrons to the conduction band but also modify the band structure [4], [5] near the Fermi level. Somewhere between $x = 0.167$ and $x = 0.2$, the Fermi level moves up into the conduction band while the density of states in the valence band is reduced and a structural phase transition occurs ($W_{20}O_{58}$ to $W_{18}O_{49}$)[6]).

Supplementary Figure 3 (c,d) show Scanning Electron Microscope (SEM) images taken in a Focused Ion Beam (FIB) system. The cross-sectional view shows the result of the Mechanical Chemical Polishing (CMP) step that was performed to remove the topography after M2. Clearly, the passivation has the same height as the W metal lines.

Supplementary Figure 3 (e,f) show a cross-sectional BF-STEM image of a crystallized HZO grain and the corresponding fast Fourier transform.

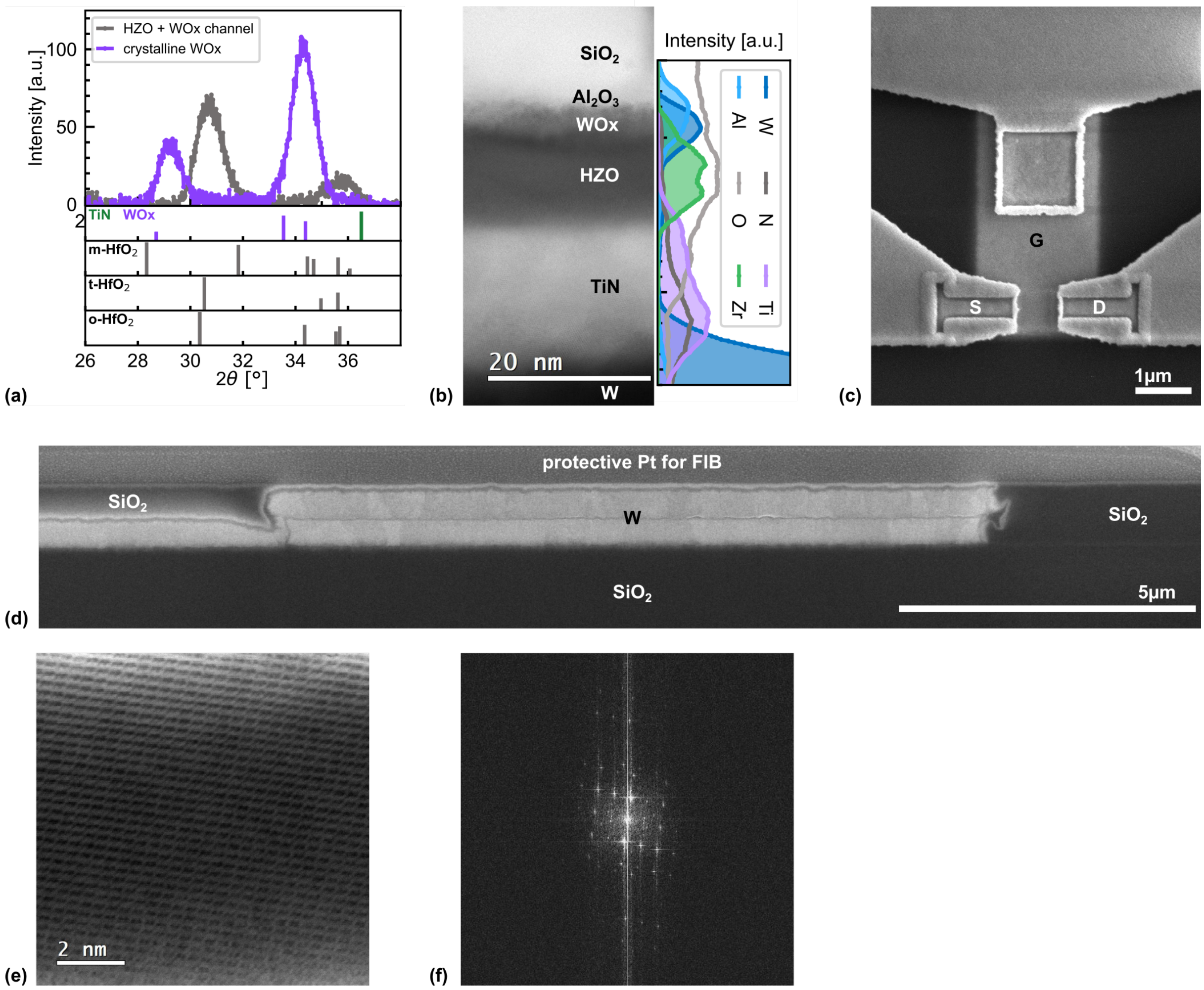

Supplementary Figure 3: Structural Analysis: **(a)** GIXRD for a diffraction angle (2θ) from 26 to 38° showing the presence of the orthorhombic/ tetragonal crystalline phase in HZO after ms-FLA crystallization and deposition of the amorphous WOx channel (gray). Crystalline WO_x for comparison (purple). **(b)** Cross-sectional BF-STEM image with energy-dispersive X-ray spectroscopy (EDS) line profile of the SiO_2/Al_2O_3/WOx/HZO/TiN/ Si gate region.
Scanning Electron Microscope (SEM) images of the fabricated FeFETs: **(c)** A top-down view showing the gate (G) coming from the top and source (S) and drain (D) form the sides. The gate extends below the channel which is situated between S and D. **(d)** Focused ion beam (FIB) cross sectional SEM image showing the result of the planarization step by chemical mechanical polishing (CMP): The topography introduced by M1 and M2 is removed. The columnar growth of the W (M1, M2) clearly introduces a roughness to the gate. (**e**) BF-STEM image of a HZO crystalline grain and **(f)** the corresponding Fast Fourier Transform.

Supplementary Note 3. Gate capacitance and resistance

Devices with a channel length and width from $300\ nm$ to $2\ \mu m$ were fabricated to capture the geometrical influence on the device performance. In our design the gate (G) fully overlaps with the source (S) and the (D). The channel length is defined as the distance between the S and D contacts. First, the gate capacitance was measured across 30 FeFETs of different sizes and normalized by the gate area: A typical butterfly shaped capacitance dependence on the gate voltage (V_G) was observed with $C_G = 24\ fF \cdot \mu m^{-2}$ at $V_G = 0V$ (**Supplementary Figure 4(a)**). This is in good agreement with previous studies[7].

The gate resistance (R_G) was measured on the same 30 FeFETs by applying a V_G from $1\ V$ to $5\ V$ while grounding source and drain (**Supplementary Figure 4(b)**). At $1\ V$ the gate resistance is $740\ G\Omega \cdot \mu m^2$, while at 5 V it decreases to $39\ G\Omega \cdot \mu m^2$. Both values are high impedance and allow for low power writing. In addition, decreasing the device size reduces the power dissipation even more.

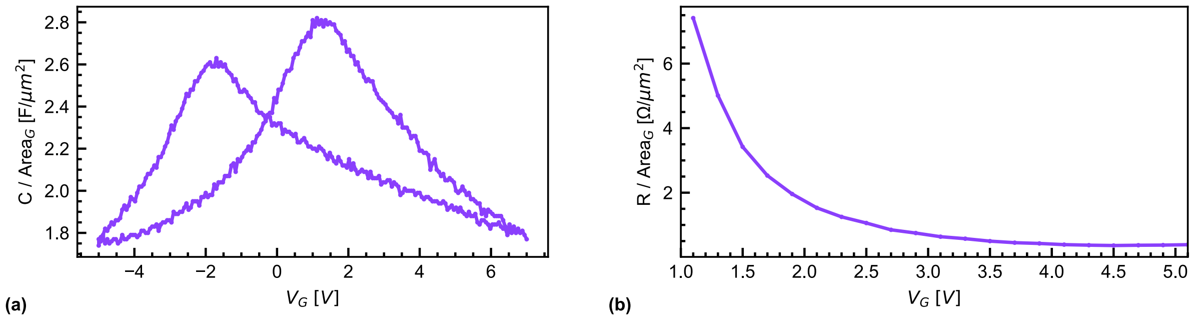

Supplementary Figure 4: Gate capacitance and resistance normalized by the gate area. Due to an overlap of S, D and G, the gate area is slightly larger than the channel area. **(a)** Average gate capacitance (C_G) and **(b)** average gate resistance (R_G) as a function of gate voltage (V_G).

Supplementary Note 4. Ferroelectric Polarization – PUND

Ferroelectric properties of the HZO layer were analyzed by measuring positive-up negative-down (PUND) characteristics on a $40x40\ \mu m^2$ TiN/HZO/WO_x/W (MFSM) capacitor on the same sample as the FeFETs (**Supplementary Figure 5**), resulting in a positive (negative) remanent polarization $+P_r$ = 17.7 µC/cm^2 ($-P_r$ = 11.2 µC/cm^2) and a positive (negative) coercive field $V_{c+} = 2.0\ V$ ($V_{c-} = 2.57\ V$). The asymmetric coercive field is due to asymmetric electrode work-functions (WO_3: 6.8 eV[8], $W_{18}O_{49}$: $6.4\ eV$[8], TiN: $4.55\ eV$[9]) that result in a build-in field. The reduced negative remanent polarization ($-P_r = 11.2\ \mu C \cdot cm^2$) as compared to the positive one ($+P_r = 17.7\ \mu C \cdot cm^2$) is an indication of partially switched domains due to incomplete screening by the depleted WO_x layer and the thereby resulting depolarization field[10] across HZO. FeFETs with low hole-density channels show partial switching due to incomplete screening (charge balance requirements[11], [12]).

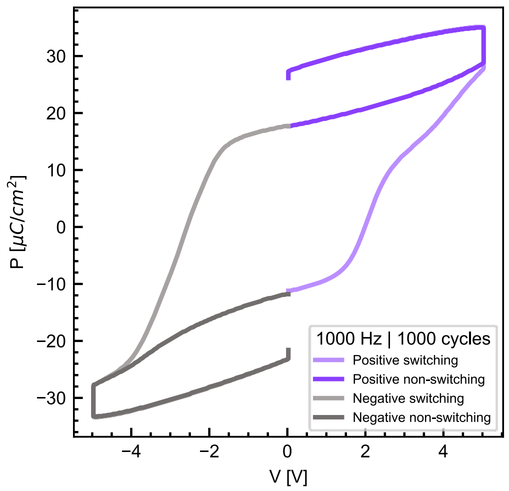

Supplementary Figure 5: Positive Up, Negative Down (PUND) measurements of a MFSM capacitor. The purple lines correspond to the positive pulses and the grey lines to the negative pulses.

Supplementary Note 5. Time and channel length dependent dynamic range

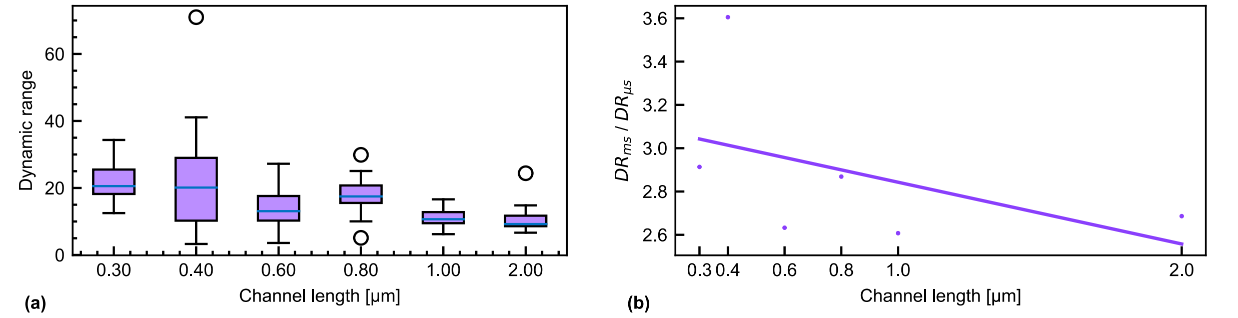

Supplementary Figure 6: Time and channel length dependent dynamic range. (a) Dynamic range (DR) measured on devices with a channel width of 600 nm and a channel length varying from 300 nm to 2 μm. Write pulses with an amplitude (V_w) from $-6\ V$ to $6\ V$ and width (t_w) of 500 μs were applied. The boxes extend from the lower to upper quartile values of the data, with a line at the median. The whiskers extend from the box to show the range of the data. Flier points are those past the end of the whiskers. **(b)** Factor by which the dynamic range increased by going $t_w = 500\ \mu s$ (**Figure 1(b)**) to $t_w = 500\ ms$ as a function of channel length. Note that the outlier with a high dynamic range at the channel length 0.4 μm increases the slope.

The infulence of the write pulse width (t_w) on the dynamic range (DR) was obtained by measuing potentiation and depression ($V_w \pm 6\ V$) on 120 FeFETs with a channel width W_{ch} of 600 nm and variyng channel length L_{ch} from 300 nm to 2 µm. Repeating the same measuremnt for a t_w of 500 μs (**Figure 1(b)**) and 500 ms (**Supplementary Figure 6 (a)**) showed a clear dynamic range increase with increasing t_w. **Supplementary Figure 6(b)** shows the factor by which the dynamic range increased by increasing t_w as a function of the channel length. There is a small dependence on the channel length.

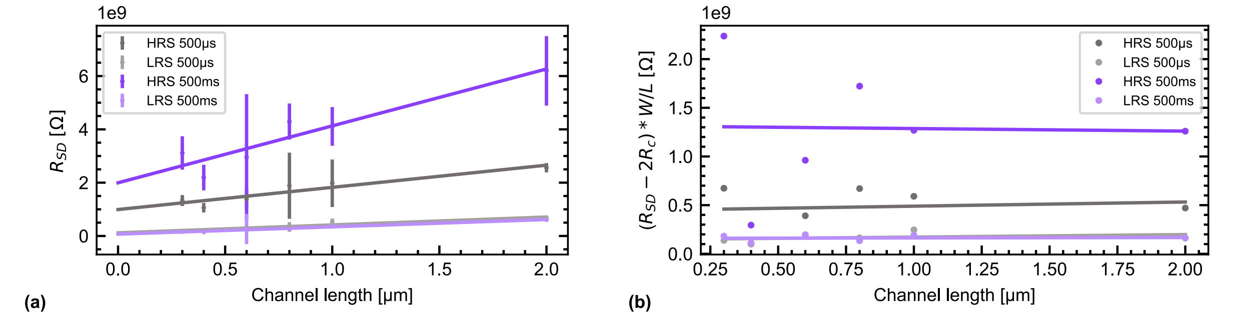

Supplementary Figure 7: Contact resistance extraction. (a) *The contact resistance at the y-intercept of the LRS and HRS as a function of the FeFET channel length. The error bars denote the standard deviation.* ***(b)*** *LRS and HRS obtained by subtracting the contact resistance and normalizing by the channel dimensions (W_{Ch}/L_{Ch}).*

We can now define the total source-to-drain resistance as $R_{SD} = R_{ch} + 2R_c$, where $R_{ch} = \frac{\rho}{t} \cdot \frac{L}{W}$ is the resistance of the WOx channel without the contact resistance (2Rc).

Plotting the LRS and HRS as a function of the channel length (**Supplementary Figure 7(a)**) is similar to transmission line measurements (TLM) and allows to extract the contact resistance ($2R_c$) at the y-intercept and the resistivity of the channel (ρ) from the slope of the linear regression. The extracted values for $t_w = 500\ \mu s$ and $t_w = 500\ ms$ are summarized in **Supplementary Table 1**. Multiple observations can be made: First, the R_c modulation has a greater influence on the DR than the ρ modulation. The same conclusion holds when comparing the DR by increasing t_w: The effect is more pronounced for R_c than for ρ.

t_{w}	$2R_{\mathrm{c}}$ (LRS)	$2R_{\mathrm{c}}$ (HRS)	$DR_{R_{\mathrm{c}}}$	ρ (LRS)	ρ (HRS)	$DR_{R_{\mathrm{ch}}}$
500 µs	$118\ M\Omega$	$1000\ M\Omega$	8.4	$71\ \Omega cm$	$200\ \Omega cm$	2.8
500 ms	$69\ M\Omega$	$2000\ M\Omega$	29	$65\ \Omega cm$	$512\ \Omega cm$	7.4
Factor ms/µs	0.58	2	3.45	0.92	2.56	2.64

Supplementary Table 1: Pulse width dependent contact resistance. Contact resistance (R_c) and channel resistivity (ρ) extracted from **Supplementary Figure 7(a)**.

Supplementary Figure 7(b) shows the LRS and HRS for $t_{\mathrm{w}} = 500\ \mu s$ and $t_{\mathrm{w}} = 500\ ms$ with $2R_{\mathrm{c}}$ subtracted and scaled by the channel dimensions as a function of the channel length ($R_{\mathrm{scaled}} = \frac{(R_{\mathrm{SD}} - 2R_{\mathrm{c}})W_{\mathrm{ch}}}{L_{\mathrm{ch}}}$). The fact that the linear regression has almost no slope shows that R_{ch} scales with the geometry of the channel and that there is very little dependence of the $DR_{R_{\mathrm{ch}}}$ on the channel length. The dependence of the DR on the channel length must originate from the difference in the ratio of $DR_{\mathrm{R_{ch}}}$ and $DR_{\mathrm{R_c}}$ for different channel lengths.

Supplementary Note 6. Oxygen movement

The ferroelectric effect is field-dependent and the polarization is supposed to saturate as soon as all domains have switched. In a FeFET this should translate into a saturation of the channel resistance as soon as the entire ferroelectric layer has switched. **Supplementary Figure 9** shows a non-saturating channel resistance when applying 90 identical pulses with $V_w = 6\ V$ and $t_w = 100\ ms$, indicating that there is a second effect like oxygen movement involved in the modulation.

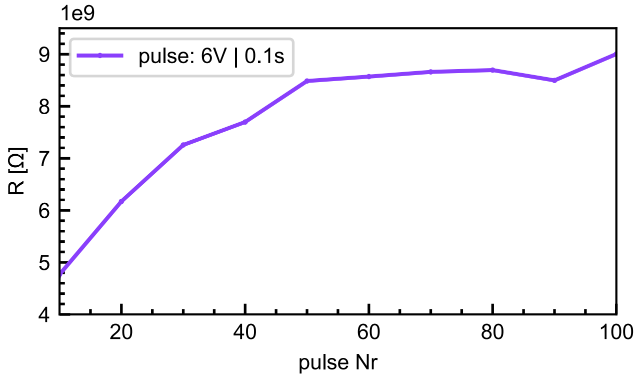

Supplementary Figure 8: Non-saturating increase of the resistance. Continuous increase of resistance by repeating applying a square pulse with an amplitude of $6\ V$ and a duration of $100\ ms$. Even after 100 pulses no clear saturation can be seen.

Supplementary Note 7. Temperature dependent conduction measurements

Temperature dependent current measurements of the channel were conducted for two reasons: on one hand to characterize the WO_x channels conduction mechanism. On the other hand, to further prove that two different effects are contributing to the resistance modulation of the channel at different timescales. The experiment was conducted as follows. At each temperature, the device was first set to its HRS, then the channel current (I_{DS}) was measured by applying an I-V sweep from $V_{SD} = -200\ mV$ to $V_{SD} = 200\ mV$. Then the same was repeated for the LRS. After the I-V measurements were conducted, the temperature was increased. To stabilize the temperature, we waited 10 min before starting with the next I-V sweeps.
The following equation describes the ohmic conduction:

$$J_{SD} = \sigma E = \mu q N_C \exp\left[\frac{-(E_C - E_F)}{kT}\right]\frac{V_{SD}}{L_{ch}}, \qquad (1)$$

$$\triangleq$$

$$Log(J_{SD}) = Log\left(\frac{\mu q N_C}{L}\right) + \frac{-(E_C - E_F)}{k} \times \frac{1}{T} + Log(V_{SD}) \qquad (2)$$

where J_{SD} is the current density, σ the electrical conductivity, μ the electron mobility, q is the electronic charge, N_C the carrier concentration, $(E_C - E_F)$ the energy difference between the conduction band and the Fermi level, k the Boltzmann constant, T the absolute temperature, V_{SD} the source-drain voltage and L_{ch} the length of the channel. **Supplementary Figure 8** shows temperature dependent current measurements of the channel. **Supplementary Figure 8(a)** shows the channel current density (J) as a Log(J)-Log($|V|$) plot for the HRS and LRS at different temperatures. Here, the HRS was set by a short pulse of $t_w = 500$ µs and $V_w = -6\ V$ to stay in a regime where the ferroelectric effect is dominant. All the currents were well fitted by a line with $slope = 1$, a characteristic of ohmic conduction. **Supplementary Figure 8(b)** shows the Arrhenius plot of the y-intercepts from the linear regression of **Supplementary Figure 8(a)** as a function of $1/T$. According to **Equation (2)** we can extract $(E_C - E_F)$ from the slope ($slope = \frac{-(E_C - E_F)}{k}$). This then allows to use **Equation (1)** to determine the μN_C product, under the assumption that μN_C is temperature independent[13]. The dynamic range became smaller with increasing temperature as shown in **Supplementary Figure 8(c).** This effect could be due to a phase transition from the orthorhombic ferroelectric phase of HZO to the tetragonal, anti-ferroelectric phase at elevated temperatures, as observed in ZrO_2[14] and Si:HfO_2[15]. The same experiment was repeated where the HRS was set by 90 pulses of $t_w = 100\ ms$ and $V_w = 6\ V$, representing a much longer timescale (**Supplementary Figure 8(d)**). As can be seen in **Supplementary Figure 9**, a complete saturation of the HRS was usually not reached. Again, the current can be fitted with a linear regression with $slope = 1$. The corresponding Arrhenius plot (**Supplementary Figure 8(e)**) is used to extract $E_C - E_F$ and μN_C. Since the HRS does not saturate, it is difficult to argue that at each temperature the same state is reached and hence also explains why the conduction in **Supplementary Figure 8(e)** does not linearly depend on $1/T$. In other words, using the same pulses to set the HRS at different temperatures will result in a different oxidation state of the WO_x for each temperature. The extracted parameters are summarized in **Supplementary Table 2** where the values for the HRS should be treated with caution as explained above. Finally, **Supplementary Figure 8(f)** shows a dynamic range that increases with temperature, exactly what we expect if the resistance modulation is dominated

by oxygen movement: the mobility of oxygen increases with temperature which leads to an enhanced oxidation and reduction of the channel.

In summary, this experiment clearly shows the ohmic nature of our WO_x channel at read voltages ($V_{SD} = \pm 200\ mV$) for both the LRS and HRS. Especially by looking at the dynamic range dependence on temperature we can further prove that two different mechanisms resistance at two different timescales modulate the channel resistance.

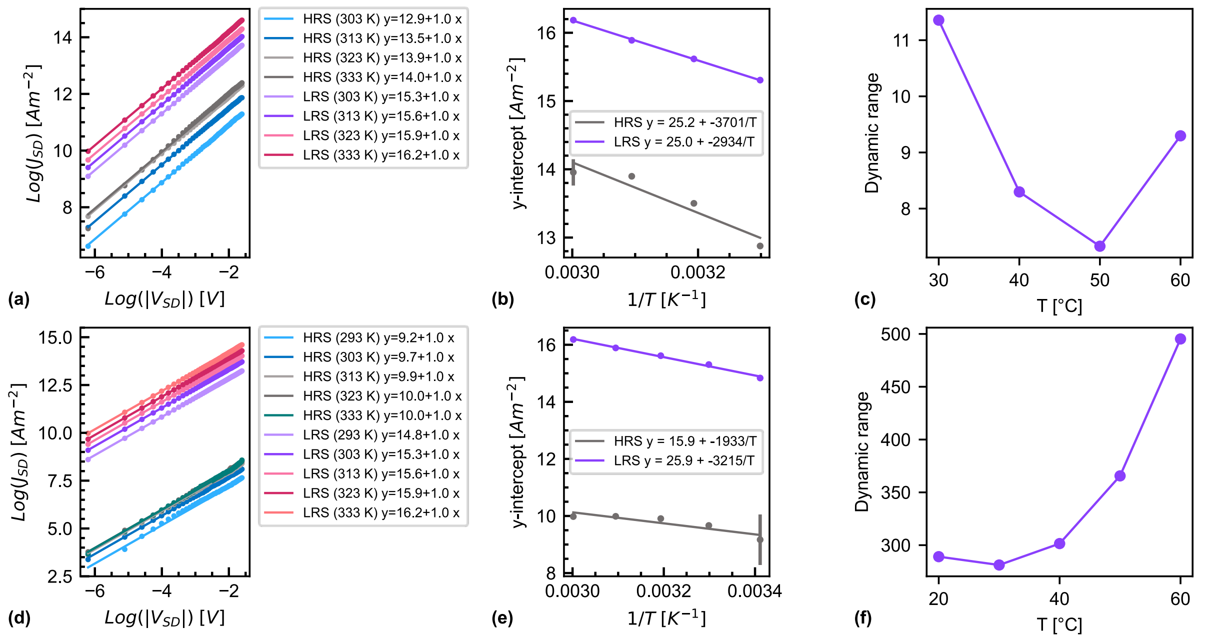

Supplementary Figure 9: Temperature dependent channel current (IDS) analysis. (a) Log(J)-Log(|V|) plot of the channel current at different temperatures for the HRS and the LRS state. The HRS was set with a short pulse of $t_w = 500\ \mu s$ and $V_w = -6\ V$. **(b)** Arrhenius plot with the y-intercept from (a) as a function of $1/T$. **(c)** The dynamic range as a function of temperature. **(d),(e),(f)** Same as (a), (b), (c) but the HRS was set by 90 long pulses of $t_w = 100$ ms and $V_w = -6$ V, trying to saturate the HRS.

	LRS (E_C-E_F)	LRS (μN_C)	HRS (E_C-E_F)	HRS (μN_C)	Dynamic range at 60°C
$t_w = 500\ \mu s$			0.32 eV	1.65e+21	9
$90 \cdot t_w = 100$ ms	0.28 eV	3.17e+21	0.17 eV	1.55e+17	500

Supplementary Table 2: Summary of the extracted values from the temperature dependent current measurements in **Supplementary Figure 9**.

Supplementary Note 8. Modification of the MLP+NeuroSimV3.0[16] code

In the original code, the conductance variation from device-to-device was equally applied to all weights in the network. This is because the random generators seed is the current time in seconds (original L3: `std::time(0)`), which produces the same output for an entire second. The initialisation of all the devices usually happens faster than a second and thus all devices get the same variation or at most two different variations. Since we want to encode a device-to-device conductance variation, the code was slightly adapted (modified L2: `rd()` instead of `std::time(0)`) to generate a random variation for each device as follows:

- L8: Calculate the dynamic range `OnOff`.
- L9-L10: Calculate the conductance variation for `minConductance` and `OnOff`.
- L13-L14: Gaussian distribution functions with `minConductanceVar` and `OnOffVar` around 0.
- L17-L20: Define min and max variations measured on samples.
- L25-L30: Draw a random sample from the Gaussian distribution function of the conductance variation.

- L31: Add the random conductance variation to the `minConductance`
- L35-L40: Draw a random sample from the Gaussian distribution function of the dynamic range variation.
- L41: Add the random dynamic range variation to the dynamic range `OnOff`.
- L43: Multiply `minConductance` with the random dynamic range to get the `maxConductance`

This way we can account for the variation in conductance and dynamic range. If a random variation from far into the gaussian tail is picked, which is higher or lower than the maximum or minimum variation measured on the devices, the process is repeated and a new variation is picked until it is within the boundaries. The resulting distributions are shown in **Figure 5**.

Original code:

```
//It's OK not to use the external gen, since here the device-to-device vairation is a one-time deal
std::mt19937 localGen;
localGen.seed(std::time(0));

/* Conductance range variation */
conductanceRangeVar = false; //Consider variation of conductance range or not
maxConductanceVar = 0; //Sigma of maxConductance variation (S)
minConductanceVar = 0; //Sigma of minConductance variation (S)
gaussian_dist_maxConductance = new std::normal_distribution<double>(0, maxConductanceVar);
gaussian_dist_minConductance = new std::normal_distribution<double>(0, minConductanceVar);

if (conductanceRangeVar) {
  maxConductance += (*gaussian_dist_maxConductance)(localGen);
  minConductance += (*gaussian_dist_minConductance)(localGen);

  // Conductance variation check
  if (minConductance >= maxConductance || maxConductance < 0 || minConductance < 0 )
  {
    puts("[Error] Conductance variation check not passed. The variation may be too large.");
    exit(-1);
  }
  // Use the code below instead for re-choosing the variation if the check is not passed
  //do {
  //  maxConductance = avgMaxConductance + (*gaussian_dist_maxConductance)(localGen);
  //  minConductance = avgMinConductance + (*gaussian_dist_minConductance)(localGen);
  //} while (minConductance >= maxConductance || maxConductance < 0 || minConductance < 0);
}
```

Modified code:

```
std::random_device rd{};
std::mt19937 localGen(rd()); //Change to rd() to create a random seed for every device

/* Conductance range variation *///=Variation of HRS and LRS from device-to-device
conductanceRangeVar = true; //Consider variation of conductance range or not
if (conductanceRangeVar)
{
  OnOff = maxConductance / minConductance;
  minConductanceVar = 0.39 * minConductance; //Sigma of minConductance variation (S)
  OnOffVar = 0.29 * OnOff; //Sigma of OnOff variation

  //create gaussian distribution function around 0
  gaussian_dist_minCond = new std::normal_distribution<double>(0, minConductanceVar);
  gaussian_dist_OnOffVar = new std::normal_distribution<double>(0, OnOffVar);

  //max and min values in all devices measured in % of the mean
  double minCond_clip_max = 1.88;
  double minCond_clip_min = 0.38;
  double OnOffVar_clip_max = 1.43;
  double OnOffVar_clip_min = 0.48;

  /*If random sample from gaussian distribution is more/less than the max/min device
   measured, repeat if this is not done, sometimes a random sample is so far in the
   tail of the gaussian, that it makes the resulting conductance negative*/
  double minCondChange = 0;
  do{
    minCondChange = (*gaussian_dist_minCond)(localGen);

  }while((minConductance + minCondChange) <= (minConductance * minCondVar_clip_min)
      || (minConductance + minCondChange) >= (minConductance * minCondVar_clip_max));
  minConductance += minCondChange;

  /*Multiply minConductance by a OnOff value from a gaussian distribution around the
  mean. Repeat if OnOff is too far in the tale*/
  double OnOffChange = 0;
  do{
    OnOffChange = (*gaussian_dist_OnOffVar)(localGen);

  }while((OnOff + OnOffChange) <= (OnOff * OnOffVar_clip_min)
      || (OnOff + OnOffChange) >= (OnOff * OnOffVar_clip_max));
  OnOff += OnOffChange;

  maxConductance = minConductance * OnOff;
  printf("conductance=%.4e / %.4e / %f\n", minConductance,maxConductance,OnOff);

  // Conductance variation check
  if (minConductance >= maxConductance || maxConductance < 0 || minConductance < 0 )
  {
    puts("[Error] Conductance variation check not passed. The variation may be too large.");
    exit(-1);
  }
}
```

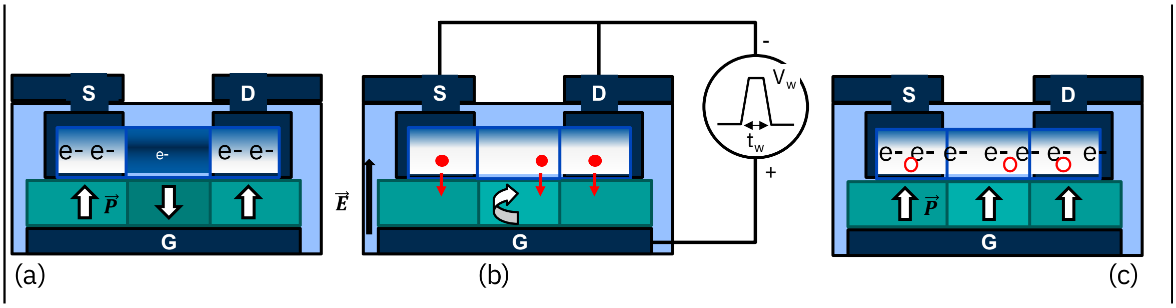

Supplementary Figure 10: Channel-resistance modulation effects. Schematic diagram illustrating the resistive switching mechanism. (a) Only electrostatic effects are represented. When the polarization points towards (resp. outwards) the n-type, WO_x channel, carriers accumulate (resp. deplete) in the channel. The electrostatic, ferroelectric field-effect is responsible of the Source (S) - Drain (D) modulation at every timescale. (b) A programming pulse of duration t_w is applied on the gate. The ferroelectric domains anti-parallel to the field switch (white arrow). If t_w is long enough, an additional mechanism occurs: the WO_x channel is reduced by the HZO (red arrows). (c) as a result, the oxygen vacancies in the WO_x channel further decrease the R_{SD} resistance. As the field is applied in the opposite direction, the oxygen atoms are pushed back from the HZO to the WO_x channel (not represented).

	FeFET (M. Jerry et al. [17])	IGZO FeFET (M.-K. Kim and J.-S. Lee [18])	Ge NW pFeFET (M. Si et al. [19])	Hybrid FeFET (X. Sun et al. [20])	SL FeFET (K. A. Aabrar et al. [21])	This work
# of conductance levels	32	64	320/256	64	128	241/217
Non-linearity	1.75/1.46	-0.8/-0.69	1.2/-1.75	0.5/0.5	-0.7/-1.56	2.3/-4.6
R_{ON} (kΩ)	560	5400	5	560	61	20000
DR	45	14	500	45	286	16
Online learning accuracy	88%	92%	86%	94%	94%	92%

Supplementary Table 3: Comparison to state-of-the-art FeFETs. Benchmarking of the proposed FeFET for ANNs. The online learning accuracy is estimated using NeuroSim MLP platform, before correction.

Supplementary References

[1] É. O'Connor *et al.*, "Stabilization of ferroelectric Hf x Zr 1−x O 2 films using a millisecond flash lamp annealing technique," *APL Mater.*, vol. 6, no. 12, p. 121103, Dec. 2018.

[2] A. Aird, M. C. Domeneghetti, F. Mazzi, V. Tazzoli, and E. K. H. Salje, "Sheet superconductivity in : crystal structure of the tetragonal matrix," *J. Phys. Condens. Matter*, vol. 10, no. 33, pp. L569–L574, Aug. 1998.

[3] R. Haubner, W. D. Schubert, E. Lassner, and B. Lux, "Influence of aluminum on the reduction of tungsten oxide to tungsten powder," *Int. J. Refract. Hard Met.*, vol. 6, no. 3, pp. 161–167, 1987.

[4] A. Georg, W. Graf, and V. Wittwer, "Comparison of electrical conductivity and optical

properties of substoichiometrically and electrochemically coloured WOx films of different crystallinity," *Sol. Energy Mater. Sol. Cells*, vol. 51, no. 3–4, pp. 353–370, Feb. 1998.
[5] O. Glember and H. Saurr, "Über Wolframoxyde," *Zeitschrift für Anorg. Chemie*, vol. 252, no. 3–4, pp. 144–159, Sep. 1943.
[6] B. Ingham, S. C. Hendy, S. V Chong, and J. L. Tallon, "Density-functional studies of tungsten trioxide, tungsten bronzes, and related systems," *Phys. Rev. B*, vol. 72, no. 7, p. 075109, Aug. 2005.
[7] M. Halter *et al.*, "Back-End, CMOS-Compatible Ferroelectric Field-Effect Transistor for Synaptic Weights," *ACS Appl. Mater. Interfaces*, vol. 12, no. 15, pp. 17725–17732, Apr. 2020.
[8] M. T. Greiner and Z.-H. Lu, "Thin-film metal oxides in organic semiconductor devices: their electronic structures, work functions and interfaces," *NPG Asia Mater.*, vol. 5, no. 7, pp. e55–e55, Jul. 2013.
[9] S. A. Vitale, J. Kedzierski, P. Healey, P. W. Wyatt, and C. L. Keast, "Work-Function-Tuned TiN Metal Gate FDSOI Transistors for Subthreshold Operation," *IEEE Trans. Electron Devices*, vol. 58, no. 2, pp. 419–426, Feb. 2011.
[10] R. R. Mehta, B. D. Silverman, and J. T. Jacobs, "Depolarization fields in thin ferroelectric films," *J. Appl. Phys.*, vol. 44, no. 8, pp. 3379–3385, Aug. 1973.
[11] M. Si and P. D. Ye, "The Critical Role of Charge Balance on the Memory Characteristics of Ferroelectric Field-Effect Transistors," *IEEE Trans. Electron Devices*, pp. 1–6, 2021.
[12] M. Si, Z. Lin, J. Noh, J. Li, W. Chung, and P. D. Ye, "The Impact of Channel Semiconductor on the Memory Characteristics of Ferroelectric Field-Effect Transistors," *IEEE J. Electron Devices Soc.*, vol. 8, no. July, pp. 846–849, 2020.
[13] S. M. Sze and K. K. Ng, *Physics of Semiconductor Devices*, vol. 10. Hoboken, NJ, USA: John Wiley & Sons, Inc., 2006.
[14] J. Müller *et al.*, "Ferroelectricity in Simple Binary ZrO 2 and HfO 2," *Nano Lett.*, vol. 12, no. 8, pp. 4318–4323, Aug. 2012.
[15] T. S. Böscke *et al.*, "Phase transitions in ferroelectric silicon doped hafnium oxide," *Appl. Phys. Lett.*, vol. 99, no. 11, p. 112904, Sep. 2011.
[16] P.-Y. Chen, X. Peng, and S. Yu, "NeuroSim+: An integrated device-to-algorithm framework for benchmarking synaptic devices and array architectures," in *2017 IEEE International Electron Devices Meeting (IEDM)*, 2017, pp. 6.1.1-6.1.4.
[17] M. Jerry *et al.*, "A ferroelectric field effect transistor based synaptic weight cell," *J. Phys. D. Appl. Phys.*, vol. 51, no. 43, p. 434001, Oct. 2018.
[18] M.-K. Kim and J.-S. Lee, "Ferroelectric Analog Synaptic Transistors," *Nano Lett.*, vol. 19, no. 3, pp. 2044–2050, Mar. 2019.
[19] M. Si *et al.*, "A Novel Scalable Energy-Efficient Synaptic Device: Crossbar Ferroelectric Semiconductor Junction," in *2019 IEEE International Electron Devices Meeting (IEDM)*, 2019, vol. 2, no. 12, pp. 6.6.1-6.6.4.
[20] X. Sun, P. Wang, K. Ni, S. Datta, and S. Yu, "Exploiting Hybrid Precision for Training and Inference: A 2T-1FeFET Based Analog Synaptic Weight Cell," in *2018 IEEE International Electron Devices Meeting (IEDM)*, 2018, vol. 2018-Decem, pp. 3.1.1-3.1.4.
[21] K. A. Aabrar *et al.*, "BEOL-Compatible Superlattice FEFET Analog Synapse With Improved Linearity and Symmetry of Weight Update," *IEEE Trans. Electron Devices*, pp. 1–7, 2022.
